# Supplementary material for: Sensitivity and specificity of commercially available rapid diagnostic tests for viral hepatitis B and C screening in serum samples
Source: PLoS One. 2020 Jul 15;15(7):e0235036. doi: 10.1371/journal.pone.0235036 (PMC7363090; doi:10.1371/journal.pone.0235036)
Supplement: Attachment — Submitted filename: Response to reviewer 07042020.docx [file pone.0235036.s001.docx]

Dear reviewer,

we would like to thank for your thorough review which helps improving the quality of this paper. We have tried to address all of the points raised. As for the inital version, it was intended as a short communications article with a strong focus on sensitivity and specificity of the RDTs. Since Plos One does not have tight restrictions regarding table numbers and word count, the manuscript has been expanded and other indicators, such as likelihood ratios have been included. Two major changes have been added to the manuscript:

Firstly, the results have been put into context for screening activities in Mongolia, which was initially the main motivation for this research. Predictive values have been calculated using the prevalence for HBV and HCV among Mongolian adults (11% and 8.5%, respectively). Applying formulas of predictive values directly to the sample of this study does not make sense, since HBV and HCV prevalence of this sample (33% each) has been created artificially by study design.

Secondly, what you raised in point 9 - effect of HBsAg positivity on specificity of HCV-Ab RDTs. This was somewhat overlooked in the initial analysis and in the revised manuscript we have explored it further. The effect has major implications on the overall conclusion of the study since false positives for HCV-Ab RDTs are more than 10 times more likely among HBsAg positives compared to controls. This has been addressed extensively in the new version.

The full raw data of this research is available under https://doi.org/10.6084/m9.figshare.9724157 . Under this link, also the extended analysis regarding HBsAg positivity and false positives for HCV-Ab RDTs can be found.

Overall we hope that we managed to improve the manuscript based on your input and we are looking forward to your answer.

Sincerely yours,

Dr. Andreas Bungert

In the following the comment by the reviewer (in italic) and our answers:

*1. Is the manuscript technically sound, and do the data support the conclusions?

The manuscript must describe a technically sound piece of scientific research with data that supports the conclusions. Experiments must have been conducted rigorously, with appropriate controls, replication, and sample sizes. The conclusions must be drawn appropriately based on the data presented. 

Reviewer #1: Partly*

*2. Has the statistical analysis been performed appropriately and rigorously? 

Reviewer #1: No*

🡪 We have included additional statistical analysis as requested by the reviewer.

*3. Have the authors made all data underlying the findings in their manuscript fully available?

The* [*PLOS Data policy*](http://www.plosone.org/static/policies.action#sharing) *requires authors to make all data underlying the findings described in their manuscript fully available without restriction, with rare exception (please refer to the Data Availability Statement in the manuscript PDF file). The data should be provided as part of the manuscript or its supporting information, or deposited to a public repository. For example, in addition to summary statistics, the data points behind means, medians and variance measures should be available. If there are restrictions on publicly sharing data—e.g. participant privacy or use of data from a third party—those must be specified.

Reviewer #1: No*

-> All underlying data and analysis is available under https://doi.org/10.6084/m9.figshare.9724157

*4. Is the manuscript presented in an intelligible fashion and written in standard English?

PLOS ONE does not copyedit accepted manuscripts, so the language in submitted articles must be clear, correct, and unambiguous. Any typographical or grammatical errors should be corrected at revision, so please note any specific errors here.

Reviewer #1: No*

🡪 We addressed problems with ambiguous expressions in the text.

*5. Review Comments to the Author*

*Please use the space provided to explain your answers to the questions above. You may also include additional comments for the author, including concerns about dual publication, research ethics, or publication ethics. (Please upload your review as an attachment if it exceeds 20,000 characters)*

*Reviewer #1: This work shows the results of the comparison of a comprehensive number of rapid diagnostic tests (RDT) for hepatitis C antibodies on a relatively modest number of samples previously characterized by ELISA (used as gold- standard).*

*MAJOR POINTS
1. L76. In the inclusion criteria, were patients with previous/current antiviral treatment included or excluded. If included, please specify treatment details.*

—> Addressed: L 146

*2. The authors use routine serology as the gold standard to evaluate the performance of RTDs. Please provide methods and kit details of the standard assays used for HBsAg and HCV-Ab testing (e.g. ELISA, MEIA, kit name, manufacturer, etc.)*

—> In the manuscript, L107

*3. For the statistical analysis, the authors used Wilson score for assessing the specificity and sensitivity of the RDTS, and this reviewer interprets that likelihood ratios were also calculated. This is not clarified in the methods, and reference 7 on likelihood tests is not referenced in the main text.*

—> The reviewer is correct, that likelihood ratios were calculated and included in an earlier version, however we had decided to take them out in favor of a shorter, manuscript with focus on sensitivity and specificity. However, we have included the values again in the results.

*4. In this line, the authors somewhat interchange concepts that are in fact different variables: test performance, sensitivity, and diagnostic accuracy. The performance is the evaluation of all variables (true positives, negatives, sensitivity, specificity, negative and positive predictive values, likelihood ratios for positive and negative results and diagnostic accuracy). Likelihood ratios and diagnostic accuracy values are not provided (see below).*

-> We have addressed this by taking chaning the wording to prevent confusion.

*5. L126 and Table 2. Can the authors provide more data on the studied cohorts, such as ALT, AST, GGT, fibrosis scores/stage of liver disease (if available), other HBV serological markers (e.g. HBeAg), risk factors, etc.?*

-> Unfortunately, we do not have these data from that time since it was considered beyond the narrow scope of the study and would have required more extensive testing.

*6. L139-143. Here there is a confusion between performance, sensitivity and accuracy (see above). Please, use appropriate terms.*

-> See point 4

*7. Table 3. I would suggest ordering the tests by sensitivity and then by specificity. Add percentages for quotients. Add columns with values for positive and negative predictive values, likelihood ratios and calculated diagnostic accuracy (including 95% CI). See examples:. Larrat, et al J. Clin. Virol. 55(3) (2012) 220–225; Cloherty, et al. J. Clin.Microbiol. 54 (2016) 265–273.*

-> Regarding the ordering by sensitivity and specificity, we have considered this. However, we have to different tests for each manufacturer and we prefer to be consistent across all tables. Therefore we opted for a neutral alphabetic order. However we have include likelyhood ratios, predictive values and diagnostic accuracy.

*8. The same applies for Table 4.*

-> See 7

*9. L158. “...in HCV-Ab negative, HBsAg positive sera than in sera from healthy...” Do the authors have any explanation for this? Include in the discussion.*

-> This is the most important point of the revision and large parts have been rewritten to address this issue. We are still trying to get some explanations by the manufacturers and also we are in discussions with other scientists regarding this issue. Currently we do not have a conclusive explanation for the effect.

*10. L160. I believe that figure 1 is redundant with the tables. The authors may want to transfer details provided in the caption to the text, or to a new table describing the details for all the discrepant results.*

-> Addressed

*11. In the discussion the authors argue about tests performance, the value of diagnostic accuracy, when this was not calculated, etc-...please, re-write the discussion section completely after the lacking variables have been analyzed*

Addressed

*12. L203-205. Delete paragraph*

Addressed

*13. L206-209. Rephrase, focus on the importance of tasting in the field with real world conditions on your environment, and what how to identify HCV-Ab positives by RDTs with negative HCV-RNA.*

We have extended our discussion section of this issue.

*14. The list of references is quite scarce, there have been a quite high number of paper published in the topic. For a reference see for instance Peeling et al. BMC Infect Dis. 2017 Nov 1;17(Suppl 1):699*

More references have been included

Minor points have been addressed
